# Application of Extracellular Vesicles in Allergic Rhinitis: A Systematic Review

**DOI:** 10.3390/ijms24010367

**Published:** 2022-12-26

**Authors:** Katarzyna Czerwaty, Karolina Dżaman, Wiktor Miechowski

**Affiliations:** Department of Otolaryngology, Centre of Postgraduate Medical Education, Marymoncka 99/103, 01-813 Warsaw, Poland

**Keywords:** allergic rhinitis, extracellular vesicles, exosomes, miRNA, LncRNA, liquid biopsy

## Abstract

The pathophysiology of allergic rhinitis (AR), one of the most common diseases in the world, is still not sufficiently understood. Extracellular vesicles (EVs), which are secreted by host and bacteria cells and take part in near and distant intracellular communication, can provide information about AR. Recently, attention has been drawn to the potential use of EVs as biomarkers, vaccines, or transporters for drug delivery. In this review, we present an up-to-date literature overview on EVs in AR to reveal their potential clinical significance in this condition. A comprehensive and systematic literature search was conducted following PRISMA statement guidelines for original, completed articles, available in English concerning EVs and AR. For this purpose, PubMed/MEDLINE, Scopus, Web of Science, and Cochrane, were searched up until 10 Novenmber 2022. From 275 records, 18 articles were included for analysis. The risk of bias was assessed for all studies as low or moderate risk of overall bias using the Office and Health Assessment and Translation Risk of Bias Rating Tool for Human and Animal Studies. We presented the role of exosomes in the pathophysiology of AR and highlighted the possibility of using exosomes as biomarkers and treatment in this disease.

## 1. Introduction

Allergic rhinitis (AR) is one of the most common diseases, affecting 10–20% of people worldwide, and there is an upward trend in the incidence of the disease [1,2]. AR is inflammation of the nasal mucosa caused by reactions to inhaled allergens. The classic symptoms of AR are nasal itching, sneezing, runny nose, and nasal congestion, but ocular symptoms are also common, including itching or redness of the eyes and tearing, or other symptoms such as palate itching or cough [3]. Although the disease is not life-threatening, it unfortunately significantly affects not only the patient’s quality of life, but also education and productivity, and is associated with substantial economic costs [4,5]. Understanding the molecular mechanisms underlying AR is crucial, as it may allow the development of new therapeutic options.

Extracellular vesicles (EVs) play an important role in the intercellular transport of proteins, lipids, and nucleic acids, thus controlling various cellular processes [6,7]. In addition to eukaryotic cells, EVs are also released by bacteria, both pathogenic and commensal [8]. Recently, attention has been drawn to the potential use of EVs as biomarkers, vaccines, or transporters for drug delivery [9]. EVs have been shown to have immunoregulatory effects in autoimmune and inflammatory diseases [10,11]. Research on exosomes is a great source of information on the pathomechanism of AR [12]. Nasal-mucosa-derived exosomes play a role in the defense mechanism of the human upper airway [13]. Exosomes have also been studied for their ability to suppress allergic respiratory diseases, and the results are promising [14,15].

In this review, we aimed to comprehensively search the literature for studies that have identified exosomal cargo present in AR patients, the utility of exosomes as biomarkers, and the treatment of AR. It allowed us to specify the current state of knowledge of the role of EVs in AR and present clinical relevance and potential for further investigations.

## 2. Materials and Methods

This systematic review was conducted according to the Preferred Reporting Items for Systematic Reviews and Meta-Analyses (PRISMA) statement guidelines [16].

### 2.1. Search Strategy

A comprehensive systematic literature search was conducted to retrieve studies from the electronic bibliographic databases PubMed, Scopus, Web of Science, and Cochrane according to a systematic predefined search strategy that included terms such as “allergic rhinitis,” “exosomes,” “exosome,” “extracellular vesicles,” “exosomal,” and “liquid biopsy.” The last search was conducted on 10 November 2022, for all databases. A detailed description of the search strategy for each database is shown in Table S1. An additional snowball search covering the bibliographies of included papers was also manually performed to identify any other relevant articles.

### 2.2. Eligibility Criteria

All citations identified for further evaluation were imported into the EndNote 20 reference management software package (Clarivate Analytics, Philadelphia, PA, USA). Duplicates were found by the EndNote software and manually by two reviewers (KC and WM). The records were then screened sequentially by title, abstract, and full text by two authors independently (KC and WM) to find good-quality articles exploring exosomes in AR. Any disagreements were resolved after discussion by a third investigator (KD). The papers that were included in the data extraction met the inclusion and exclusion criteria (Table 1). No restriction was placed on the date of publication and both animal and in vitro studies were included. Review papers, conference reports, case reports, expert opinions, letters to the editor, book chapters, and unpublished articles were excluded.

### 2.3. Data Extraction

Two investigators (KC and WM) independently filtered included studies considering the following study data: first author, year of publication, country of origin, study type, purpose, main study steps, main results, source of exosomes, methods of their isolation and characterization, exosomal markers, and cargo. Additionally, in studies involving human subjects, information was collected on who constituted the study group and who constituted the control group, the sizes of these groups, and the inclusion and exclusion criteria of the study group.

### 2.4. Quality Assessment

The risk of bias in each study was assessed according to the modified Office and Health Assessment and Translation (OHAT) Risk of Bias Rating Tool for Human and Animal Studies (https://ntp.niehs.nih.gov/ntp/ohat/pubs/riskofbiastool_508.pdf, accessed on 26 November 2022) by two independent investigators (KC and WM) and disagreements were resolved by discussion by the third researcher (KD). The summarised quality assessment for every single study is reported in Table S4.

## 3. Results and Discussion

### 3.1. Study Selection, Characteristics, and Quality

**Figure 1:**
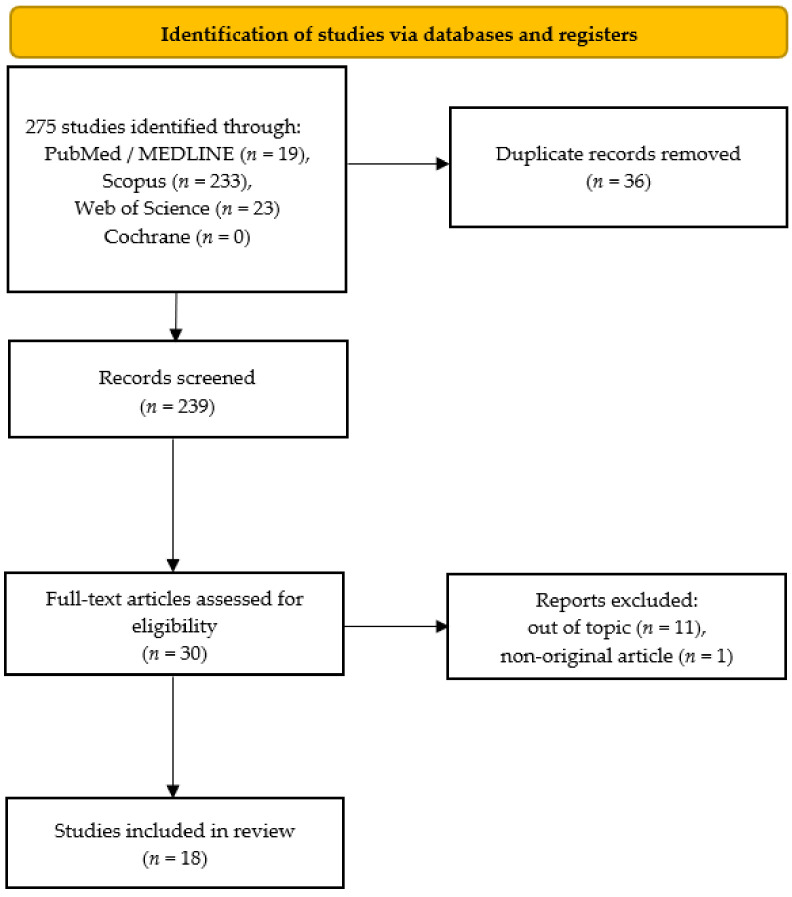
Flowchart of study selection.

The detailed selection strategy of the papers for this systematic review is shown in the PRISMA flowchart (Figure 1). 

The literature search resulted in a total of 275 records, of which 36 were duplicates, leaving 239 studies. Screening of these by titles and abstracts resulted in the retention of 30 studies. Finally, after reading full texts, 18 articles were accepted by the reviewers for final inclusion [17,18,19,20,21,22,23,24,25,26,27,28,29,30,31,32,33,34]. Eleven articles were out of topic because they did not provide information about exosomes or there were no allergy models [35,36,37,38,39,40,41,42,43,44]. One article was not original [45], because it contained the same data as Qiu’s earlier study [34] that was included in this systematic review.

**Figure 2:**
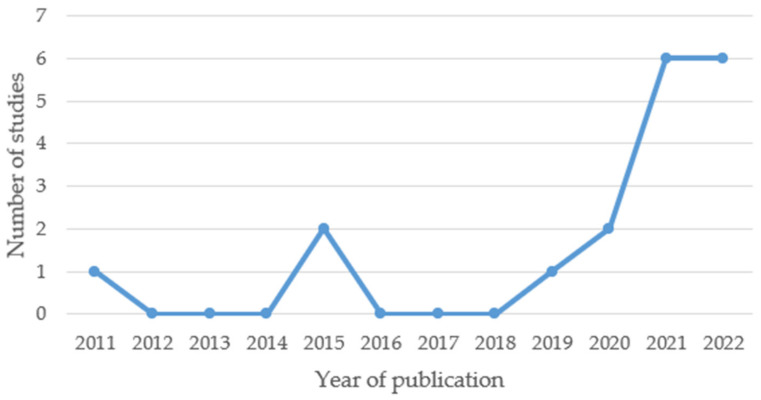
Years in which the included studies were published.

Data were collected in four different countries and as many as fourteen studies were conducted in China [17,18,20,21,22,23,24,26,28,29,30,32,33,34], two in Korea [25,31], one in Japan [19] and only one in Europe—Italy [27]. The oldest study included in this review was published in 2011, but the majority of studies were conducted in the last two years, which is presented in Figure 2 and confirms the rapid development of knowledge in the field of exosomes in recent years.

Of the 18 retrieved studies, 5 included in vivo components and were studies with an animal model (mouse) [17,19,26,30,33], 12 studies included in their structure experiments conducted in vitro using primary or established cell lines [17,18,19,20,23,24,26,28,29,30,33,34], and 6 were cross-sectional studies [21,22,25,27,31,32]. The main information on the included studies, covering the year of publication, country of origin, type of study, the purpose of the study, main steps of the methodology, and main results, are included in Table S2. 

Data extracted from people came from more than 800 participants (of which 460 were AR patients). Information on the participants of each included study, as well as on the inclusion and exclusion criteria of the study groups, are presented in Table S3.

A detailed assessment of the risk of bias for each study is shown in Table S4. All included studies were rated at low or moderate risk of overall bias.

### 3.2. Overview of Extracellular Vesicles Sources, Isolation, and Characterization Methodology

In recent years, there has been a surge in exosome research; therefore, guidelines have been established to introduce consistent principles for the isolation, purification, and characterization of EVs [46]. Highlights of the methods used for the exosomes covered in the included studies are shown in Table 2, which provides summary information on the isolation, analysis methods, markers, and cargo of exosomes. In the included studies, exosomes were isolated from various body fluids, i.e., plasma [21,27,28], urine [25,31], and nasal mucus [22,24,28,32] collected using merocele nasal tampons inserted into the inferior meatuses. However, not only body fluids were the source of exosomes, but also human or animal nasal mucosa [33,34] or cell cultures, i.e., epithelial cells (RPMI 2650, A549) [29,33,34], mesenchymal stromal cells (MSCs) [18,20,23,30], bone-marrow-derived dendritic cells (BMDCs) [26], T cells [26], follicular helper T cells (Tfhs) [17], human embryonic kidney 293 (HEK293) cells [19], and fibroblasts [30].

Isolation and purification methods were heterogeneous, but the most frequent was differential ultracentrifugation, proposed by Thery [47], to which some of the authors of the included papers referred [18,28,29,30]. The method has several steps. In the beginning, large dead cells and large cellular debris are eliminated by successive centrifugation at increasing speeds. At each stage, the pellet is discarded, and the supernatant is used for the next stage. The final supernatant is then ultracentrifuged to precipitate small vesicles that correspond to exosomes. Subsequently, the pellet is washed in a large volume of phosphate-buffered saline (PBS) to eliminate contaminating proteins, and finally, centrifuged one last time at the same height. Interestingly, there was a comparison of two methods for isolating MSC-EVs: differential ultracentrifugation [47] and anion-exchange chromatography. Compared to ultracentrifugation, exchange chromatography had some advantages: more EVs obtained (1.74 × 10^9^ vs. 9.58 × 10^8^ per 10^6^ MSCs), shorter isolation time (3 h vs. 15 h to process 600 mL of supernatant), lower cost, higher reproducibility, scalable possibility, no specialized equipment needed, and less damage to EVs [30].

Methods for analyzing exosomes also varied, but transmission electron microscopy (TEM) [17,18,19,20,21,22,23,24,26,27,28,29,30,34] and Western blotting (WB) [18,19,20,23,24,26,28,29,30,33,34] were used most often. Exosomal markers are used for exosomal characterization. The presence of positive markers and the absence of negative markers confirm that the molecules found are exosomes. The most frequently used positive exosomal markers included CD9, CD63, CD81, Alix, and TSG101, while negative exosomal markers were, unfortunately, generally not reported (NR), with only four papers pointing to calnexin [18,19,29,30] and one to GRP94 [20].

Information on the storing method of exosomes was provided only in three included studies [21,23,28], and it was freezing at −80 °C.

The researchers described EVs as spheroid, ovoid, or small cup-shaped structures [20,21,22] with diameters ranging from 30 to 150 nm under TEM [18,20,24,29], which corresponds to the definition of exosomes [6]. There were only small differences in the size and morphology among all the EVs groups.

### 3.3. The Effect of Allergic-Rhinitis-Derived Exosomes on the Occurrence of Allergic Rhinitis

The effect of AR-Tfh-derived exosomes was explored in an in vivo study on a murine model [17]. Four groups of mice were analyzed. The first was given Tfh-derived exosomes extracted from AR, the second Tfh-derived exosomes extracted from healthy mice, the third was treated with ovalbumin (OVA), and the fourth was the control group. As a result, the extent of mucosal thickening and nasal mucosal inflammation was more severe in the AR-Tfh-exosomes group and the OVA group than in the controls. The expression levels of CD80 and CD86 were the highest in the AR-Tfh-exosomes group. Moreover, in this group, the serum level of IL-13 and total IgE was significantly higher than in the control group, whereas the levels of interferon-γ (IFN-γ) and IL-17 were significantly lower. Those results indicate that AR-Tfh-derived exosomes promote Th2-type inflammation [17].

Similar conclusions come from human studies [28]. Studying antigen presentation in AR, there were found two antigens significantly implicated in allergy presented on EVs in the plasma of AR patients. One of them was Derp 1 (*Dermatophagoides pteronyssinus* protease) and the second was HLA-DR (Human Leukocyte Antigen—DR isotype)—a representative marker of APCs. Derp 1 levels on plasma EVs were strongly associated with symptom scores and type 2 cytokines (IL-13). Additionally, Derp 1 was also presented on EVs in nasal secretions of AR patients [28].

Studying the effect of EVs on the development of AR, the authors hypothesized that APCs may release EVs into circulation and mediate allergic reactions. To support this hypothesis, the authors investigated the expression of Derp 1 and molecules associated with EV antigen presentation in the plasma of house-dust-mite (HDM) -sensitive AR patients. As expected, Derp 1 was significantly higher on moderate–severe AR-EVs than on mild AR-EVs and EVs collected from healthy controls. Additionally, there was also detected the expression of costimulatory and antigen-presentation-related molecules [28]. To sum up, it has been revealed that plasma EVs may be a novel factor for Th2 differentiation in circulation during AR pathogenesis and act as mini-APCs in antigen-specific immune responses [28].

Furthermore, exosomes derived from nasal epithelial cells, collected from patients diagnosed with atypical AR, were found to promote antigen-specific differentiation of CD8+ cells [34]. Upon exposure to a specific antigen Derp 1 in the AR group, there was observed a significantly higher increase in CD 8+ T cells in nasal mucosa compared to the controls. The results indicate that antigen-specific CD8+ T cells are abundant in the nasal mucosa of patients with atypical chronic AR and are important in the pathogenesis of this disease [34].

In turn, it was indicated that the imbalance of helper T cell differentiation is involved in the development of AR [29]. Recent studies revealed the regulatory function of exosomes on Th1/Th2 differentiation [56]. However, the key mediator in exosomes that modulate such response remains unclear. Some authors [29] emphasized that it could be the exosomal long non-coding RNA GAS5 (LncGAS5) that affects the balance of Th1/Th2 differentiation and, thus, the occurrence of AR.

To sum up, most researchers confirmed that exosomes derived from AR patients influence Th1/Th2 differentiation, promote Th2-type inflammation, and are strongly associated with AR symptoms. 

### 3.4. Differentially Expressed MicroRNA in Allergic-Rhinitis-Derived Exosomes and Corresponding Biological Pathways

Various miRNAs have been investigated as biomarkers or treatment targets in AR, such as miR-124-3p [57,58], miR-3935 [37], miR-223-3p [59], miR-202-5p [60], miR-487b [61], miR-205-5P [62], miR-150-5p [63], miR-214-3p [64], miRNA-126 [65], or miR-146a [66].

In nasal-mucus-derived EVs, exactly 35 miRNAs were found differentially expressed in AR patients compared to healthy subjects (HS), of which 21 were upregulated and 14 were downregulated [32]. Subsequently, those results were validated using the TaqMan real-time qPCR method which confirmed that miR-30-5p, miR-199b-3p, and miR-203 were significantly upregulated and miR-874, miR-28-3p, and miR-875-5p were significantly downregulated in EVs from nasal mucus of AR patients compared to HS. Taking under consideration the role of those miRNAs in the signaling pathway, the authors concluded that most important in AR pathophysiology were the B-cell receptor signaling pathway, natural killer cell-mediated cytotoxicity, T cell receptor signaling pathway, the RIG-I-like receptor signaling pathway, the Wnt signaling pathway, endocytosis, and salivary secretion [32].

On the other hand, RNA was extracted from Tfhs-derived exosomes, and miR-142b and miR-142-5p were downregulated in exosomes from the AR group, compared to the control group [17]. The authors demonstrated that miR-142-5p decreased the proportion of CD80 and CD86 DCs and its inhibitor significantly increased this proportion, which suggests that exosomal miR-142-5p is involved in the regulation of DC maturation [17]. Additionally, a strong negative correlation was identified between miR-142-5p and CDK5 expression and suggested that CDK5 mediates the regulation of DC maturation by miR-142-5p [17]. 

In turn, the effect of miR-146a-5p in MSC-EVs was examined on allergic airway inflammation in mice [30]. The authors showed that miR-146a-5p antagonism in MSC-EVs abolished their immunoregulatory effect in Th-2 allergic airway inflammation and the transfer of miR-146a-5p in MSC-EVs to Th2 cells was partially associated with the therapeutic effects in Th2-dominant allergic airway inflammation. These results are in line with data obtained in another study [33], which indicate that the epithelial cell-derived miR-146a can induce IL-10 expression in CD14+ CD16− monocytes. IL-10 is an immune suppressive cytokine that can be generated by many cells. They demonstrate the functional facet of miR-146a, which contributes to immune regulation by inducing monocyte regulatory fractions and is required in maintaining functional homeostasis of regulatory T cells (Tregs) [33]. Deficiency of miR-146a results in fatal IFN-γ-dependent immune-mediated lesions in a variety of organs [67]. 

In conclusion, recent research indicates that miR-146a can induce IL-10-producing monocytes to suppress the skewed Th2 polarization, suggesting that miR-146a has potential in the treatment of allergic disorders, such as AR [33].

### 3.5. The Role of Long Non-Coding RNA in the Pathogenesis of Allergic Rhinitis 

Long non-coding RNAs (LncRNAs) are non-coding transcripts more than 200 nucleotides long, with one or more short open reading frames, which encode functional micropeptides [68]. The function of LncRNAs is mediated through interactions with both miRNAs and mRNAs and has been implicated in cell differentiation, response to stimuli, and regulation of the immune response [69,70].

Recently, LncRNAs were investigated as biomarkers in AR [36], because some of them, for example, LncRNA ANRIL, was found to be overexpressed in patients with AR [71]. Moreover, it is hypothesized they could play a therapeutic role in AR by their impact on regulating genes [72,73]. It was observed that some LncRNAs caused inhibition of granulocyte–macrophage colony-stimulating factor (GM-CSF), eotaxin, and MUAC5AC in nasal epithelial cells by influencing IL-13 production [74]. 

It was demonstrated that fragments of non-coding RNA-Lnc00632—carried by some exosomes (HUC-MSC-EVs—human-umbilical-cord-mesenchymal-stem-cell-derived extracellular vesicles) are responsible for the suppression of Th2 differentiation in CD4+ T cells [20]. Transfection of the CD4+ T cells with ov-Lnc00632 (overexpressed vectors Lnc00632) resulted in AR patients in a reduction in Th2 cells, and a drop in IL-4, and IL-13 levels. Additionally, Lnc00632 suppressed the GATA-3 (GATA binding protein-3) expression by interacting with EZH2 (enhancer of zeste homolog) [20] which is known to be important in regulating ASC function and metabolism, and also plays role in T cell differentiation [75,76].

The role of other exosomal LncRNAs in AR pathogenesis was observed as well. A close correlation was noticed between LncRNA NEAT1 (Nuclear Paraspeckle Assembly Transcript 1) and the severity of AR and its overexpression in this group [77]. Upregulated NEAT1 levels were also demonstrated in the AR cell model established by human nasal epithelial cells (HNECs) treated with IL-13. Moreover, the authors presented that the NEAT1 knockdown inhibited the production of pro-inflammatory cytokines and mucus. Subsequently, the researchers demonstrated that extracellular NEAT1 was secreted by packaging into exosomes. Finally, they presented data suggesting that NEAT1 may act in AR through the miR-511/NR4A2 (Nuclear Receptor Subfamily 4 Group A Member 2) axis. Downregulation of miR-511 promoted inflammatory cytokines, mucus production, and apoptosis in IL-13-induced HNECs, while the knockdown of NR4A2 reversed these effects. These findings point to a potential therapeutic target in patients with AR, which is NEAT1 [24]. 

On the other hand, it was demonstrated that the next LncRNA–LncGAS5 was upregulated in AR epithelial samples and exosomes isolated from nasal mucus samples of AR patients and OVA-induced RPMI 2650 cells. They showed that LncGAS5 suppresses Th1 differentiation and promotes Th2 differentiation via the regulation of EZH2 and T-bet [29].

### 3.6. The Role of Exosomes in Regulation of Dendritic Cell Maturation in Allergic Rhinitis 

Tfhs are a specialized CD4+ T cells subpopulation that play an important role in humoral immunity in allergic diseases by controlling immune responses to allergens [78,79]. Some authors observed the role of exosomes in the regulation of DC maturation in the murine model of AR [17]. Others [18] explored the influence of MSC-EVs (EVs derived from mesenchymal stromal cells) on immature DCs (iDCs). In an in vitro study, they noticed that MSC-EVs do not affect DC differentiation from monocytes at the beginning, but inhibit the differentiation of iDCs [18] and MSC-EVs did not have an influence on the expression of maturation markers from iDCs to mature DCs (mDCs) [18]. These results were consistent with other authors [80].

In turn, in another study [34] it was investigated whether DCs capture exosomes and what effect exosomes carrying Derp 1 and Staphylococcal enterotoxin B (SEB) have on DCs. The data showed that exposure to exosomes carrying SEB/Derp 1 significantly increased the levels of CD80, CD86, and MHC-I.

In line with the studies above, the exosomes impact on DCs differentiation was confirmed based on adipose-stem-cell-derived EVs [43].

### 3.7. Extracellular Vesicles for Therapeutic Applications in Allergic Rhinitis

#### 3.7.1. The Potential of Mesenchymal-Stromal-Cell-Derived Extracellular Vesicles Therapy in Allergic Rhinitis

In recent times, the therapeutic potential of MSCs in many diseases is being extensively investigated [81,82,83,84,85,86]. MSCs play the immunomodulatory role via releasing various mediators, including exosomes [87], which was also demonstrated in AR [88] and allergic airway inflammation [35]. In a murine model, treatment with MSCs derived by induced pluripotent stem cells (iPSC-MSCs) resulted in the reduction in serum levels of IgE and Th2 cytokines (IL-4, IL-5, and IL-13) in bronchoalveolar or nasal lavage fluids [89] and was also effective in neutrophilic airway inflammation, causing a reduction in Th12 cells, IL-12A, and p-STAT3 levels [90]. As mentioned above, similar conclusions were drawn in another study [64], which indicated that MSC-EV treatment resulted in the suppression of type 2 immune response [64].

Taking into consideration the potential MSC-EV therapy, the biggest challenges seem to be a huge number of homogeneous MSCs and a scalable isolation protocol for a large preparation of MSC-EVs [30]. Standardized EV isolation protocols in the Fang et al. [30] study would overcome these limitations. To minimize differences between various MSC formulations, they selected highly proliferative iPSC-MSCs as cell sources that were greatly expanded to create a cell bank for the production of iPSC-MSC-EVs. Additionally, they isolated iPSC-MSC-EVs from supernatants using anion exchange chromatography, which was more efficient and could easily be scaled to an industrial scale for future clinical applications [30].

#### 3.7.2. Extracellular Vesicles Containing T Cell Activators as Treatment of Allergic Rhinitis

It has been confirmed that some immune-competent cells such as DCs and B lymphocytes secrete exosomes that contain antigens, enabling them to contact T cells [26,91]. Based on this knowledge, there were created BMDCs-derived EVs containing two types of T cell activators—FLLL31 (tetramethylcurcumin, a curcumin analog) and complex of MHC-II and OVA (a specific antigen used in the treatment of experimental AR), named OFexo [26]. It was demonstrated that OFexo could recognize and efficiently activate OVA-specific CD4+ T cells and induce antigen-specific Tr1 cells leading to AR suppression [26]. Another study [40] showed that airway epithelial cells express CD83, and by administration of exosomes containing CD83/OVA, they inhibited airway allergy.

#### 3.7.3. Allergen-Specific Immunotherapy—Antigen-Loaded Extracellular Vesicles as Therapeutic Method for Allergic Rhinitis

Allergen-specific immunotherapy (AIT) is the only treatment for AR that targets the underlying pathophysiology and has a disease-modifying effect [92,93]. Its effectiveness and safety have been proven in many studies [56,94,95,96]. A lot of methods are investigated to improve the effectiveness of treatment, using molecules modifying the specific immunological response to allergens such as CpG DNA and OVA [97,98]. Unmethylated CpG DNA is a pattern recognition receptor (PRR) agonist that induces a strong Th1 response and interacts with TLR9 [98]. Co-culture of DCs with CpG-OVA-EVs resulted in higher concentrations of TNF-α and IL-6 [19].

Furthermore, an AR mouse model treated intranasally with CpG-OVA-EVs showed a statistically significant reduction in serum IgE levels and allergic symptoms such as rubbing and sneezing. Moreover, the mouse nasal sections showed less infiltration of inflammatory cells and thinner respiratory epithelium in the CpG-OVA-EV-treated group [19]. 

Other researchers also investigated nasal drops of exosomes in AR therapy [33]. Luo et al. treated the AR mice model and the naïve mice with nasal drops containing the miR-146a-laden exosomes for one week and compared results to the control group. Treatment with miR-146a-loaded exosomes markedly increased the frequency of IL-10+ in the nasal mucosa of both naïve and AR mice, which was not the case when mice were treated with nasal drops containing exosomes without miR-146a [33]. The therapy dramatically suppressed the skewed Th2 polarization in the AR mice. 

#### 3.7.4. Exosomal Biomarker for Predicting the Response to Immunotherapy in Allergic Rhinitis

AIT may be carried out subcutaneously (SCIT) or sublingually (SLIT) and is recommended in both seasonal and perennial AR [93]. SCIT is safe [99] and more effective than SLIT in AR children [100], but less cost-effective compared to SLIT [101]. Despite a large number of clinical trials on AIT, there is still a need to determine biomarkers of its efficiency [102]; therefore, a few investigators focused on using exosomes as markers of AIT effectiveness. 

The miRNAs in serum-derived exosomes of AR children were investigated and it was noticed that hsa-miR-4669 was significantly downregulated in the effective group [21]. Moreover, it was demonstrated that the hsa-miR-4669 level correlated with the visual analog scale (VAS) and total nasal symptoms score (TNSS), and was an independent factor associated with SCIT efficiency in AR children, which suggests that exosomal hsa-miR-4669 could be a biomarker predicting the response to SCIT (AUC = 0.785) [21].

### 3.8. Microbiota in Extracellular Vesicles from Allergic Rhinitis Patients

#### 3.8.1. Microbiota Characteristics in Nasal Extracellular Vesicles in Allergic Rhinitis

In recent years, special attention has been paid to the role of the microbiome in allergic diseases, as it is believed that it can modulate the immune response [103] and could be potentially used as a biomarker for AR diagnosis [104]. It appears that the composition of the nasal cavity microbiome may be important in the pathogenesis of AR [105]. Nasal microbiota play a key role in the development of mucosa-associated lymphoid tissue, which is important in both allergic and non-allergic inflammation [106]. The microbiome composition and diversity in nasal EVs were investigated using 16S rRNA sequencing to find differences between AR patients and HS [22]. There was found a reduced alpha diversity in nasal EVs of AR patients compared to HS, which is an indicator of the abundance and diversity of microbes. Interestingly, decreased diversity of nasal microbiome was also observed in chronic rhinosinusitis (CRS) [107,108,109] or asthma [110], which suggests that it has meaning in the pathophysiology of those diseases. The dominant bacterial classes in the nasal EVs were Alphaproteobacteria, Gammaproteobacteria, and Betaproteobacteria for both AR and HS. In AR patients, Rhodocyclales were less abundant compared to HS. The authors also used the Kyoto Encyclopedia of Genes and Genomes (KEGG) database to analyze microbial metabolic pathways in the nasal EVs and found 35 metabolic pathways that were significantly different between AR and HS, 25 of which were upregulated (including valine, leucine, and isoleucine degradation; tryptophan metabolism; toluene degradation) and 10 were downregulated (including selenocompound metabolism; protein kinases; prenyltransferases) in AR [22]. Additionally, different effects of particulate matter (PM) exposure on EVs release and nasal microbiome composition were found in AR and HS groups [27].

#### 3.8.2. Urine-Bacteria-Derived Extracellular Vesicles in Allergic Rhinitis

Urinary EVs are relatively easy to obtain and have potential applications for determining biomarkers not only related to the urinary tract, but also neurological diseases such as Parkinson’s disease [111]. 

In a study [31] where DNA was extracted from urinary-bacteria-derived EVs from AR, chronic rhinitis, atopic asthma patients, and HS, the authors observed a significantly higher Chao-1 richness index in the group of AR children compared to HS, which was also richer than in other study groups. They presented that specific bacterial taxa were correlated with CRS, AR, or asthma, and could be found in urinary EVs, which suggests that it can be a potentially useful indicator for assessing those diseases [31]. 

In another study [25], urinary EVs were analyzed in the allergic airway group, atopic control group, and HS. In the allergic airway group, three pathogenic genera (*Escherichia-Shigella*, *Klebsiella*, and *Haemophilus*) were significantly more abundant and positively correlated with total IgE and percentage of eosinophils [25]. 

The above studies demonstrate the use of EVs from accessible, easily collected biological material such as urine in uncovering the relationship between microbiota and allergic diseases. 

### 3.9. Discussion—Strengths and Limitations of This Systematic Review and Included Studies

To the best of our knowledge, this is the first systematic review describing the role of exosomes in AR. In recent years, interest has grown significantly regarding the mechanisms and role of EVs in various diseases, including AR, because EVs play a key role in intercellular communication. 

In this systematic review, we presented the current knowledge of the participation of exosomes in the pathophysiology of AR, summarized the underlying mechanisms, and highlighted the possibility of using exosomes as biomarkers in this disease and their potential use in treatment. We also introduced information about research related to the role of microbiota in AR, studied in both nasal and urine-derived exosomes. The main strength of this review is the systematic approach to identifying all research on the topic of EVs and AR. We have also included an assessment of the quality of the included studies. 

Concurrently, we also note the limitations of this systematic review. The included studies examined exosomes of various origins, both human and bacterial, which were isolated from various body fluids, secretions, and cell lines. Different methods of exosome isolation and various bioinformatics tools were used to analyze the resulting data. Due to the wide variety of included studies, we could only provide a narrative summary of the obtained data. The results of in vitro studies or in vivo studies on animals must be interpreted with caution as they do not always coincide with clinical trials on humans. Additionally, the cross-sectional character of some of the included studies [21,22,25,27,31,32] implies that there is no way to determine the cause-and-effect relationship of the correlations found. The results of the studies included in this review are limited by the relatively small numbers of patients sampled for the studies, allowing only qualitative analysis. The vast majority of patients included in the analysis are Asian, so it cannot be ruled out that the results of studies on other populations would differ, due to genetic considerations. Studies often did not include differentiating allergies in terms of the allergens causing them, which could affect the results. 

In the studies [25,31], it was not verified that patients had not taken antibiotics several weeks before the urine sample was collected. Additionally, the effect of other drugs on urine-derived EVs, especially anti-allergic drugs, cannot be ruled out [25,31]. It seems that the simultaneous collection of gut, respiratory, and urine samples would better define the role of these diagnostic methods [25,31]. Marino’s study [27] covered a small population which may have resulted in not discovering all the relationships between PM effects and concentrations of plasmatic EVs and the bacterial nasal microbiome. Additionally, determining PM exposure based on participants’ home addresses is not a fully reliable method, as it does not take into account the actual daily location and PM levels [27]. The choice of the control group in some studies, which consisted of patients with nasal cancer [34], was also not obvious to us, even taking into account that tissue without visible tumor infiltration was taken from them.

## 4. Conclusions

In conclusion, in recent years, knowledge of EVs has expanded significantly to also include AR topics. A growing body of evidence suggests that various exosome-dependent mechanisms play an important role in the pathogenesis of AR and can be used as biomarkers in this disease. This review provides information on the latest developments from preclinical studies of exosome therapy in AR, which may lay the groundwork for future research and further development of AR treatment.

## Figures and Tables

**Table 1:** Inclusion and exclusion criteria of included studies.

Inclusion Criteria	Exclusion Criteria
Studies concerning EVs and AR	Studies not related to EVs and AR
Original articles	Review papers, letters to the editor, conference reports, case reports, book chapters, expert opinions
Completed, published	Unfinished, unpublished
Full text available in English	Language other than English or only abstract available in English
Good-quality studies	Poor-quality studies

**Table 2:** Exosome information in the included studies.

Study	Source of Exosomes	Methods of Isolation		Methods of Analysis	Positive Exosomal Markers	Negative Exosomal Markers	Cargo
Teng [17]	Tfhs	ultracentrifugation		TEM, NTA	NR	NR	miR-142-5p
Peng [18]	MSCs	refer to Fang, 2020 [30]		TEM, NTA, WB	CD9, CD63, CD81, Alix, TSG101	calnexin	NR
Liu [19]	HEK293 cells	differential ultracentrifugation: 300× *g*, 10 min; 2000× *g*, 20 min; 10,000× *g*, 30 min; filtration using 0.2 µm syringe filters; 100,000× *g*, 1 h		TEM, DLS, WB	Alix, HSP70, CD81	calnexin	NR
Li [20]	MSCs	differential ultracentrifugation: 12,000× *g*, 45 min, 4 °C; resuspending in PBS; filtration using 0.22 µm filter; 110,000× g, 70 min, 4 °C		TEM, DLS, WB	CD63, CD81	GRP94	Lnc00632
Jiang [21]	serum	centrifugation (3000× *g*, 10 min) miRCURY Exosome Serum/Plasma Kit		TEM, DLS	NR	NR	hsa-miR-4669 (a total of 812 miRNAs)
Chiang [22]	nasal secretions	differential ultracentrifugation: 3000× *g*, 10 min [48]; 2000× *g*, 30 min, 4 °C; 10,000× *g*, 45 min, 4 °C; filtration using 0.45 µm syringe filter; 100,000× g, 70 min, 4 °C; resuspending in PBS; 100,000× g, 70 min, 4 °C		TEM, FC, DNA extraction	CD9, CD81	NR	microbiome
Zhou [23]	MSCs	ExoQuick precipitation kit [49] gradient centrifugation: 70,000× *g*, overnight, 4 °C; twice, 500 × *g*, 10 min; twice, 2000× *g*, 15 min; twice, 10,000× g, 30 min; 70,000× *g*, 1 h, 4 °C; resuspending in PBS; 70,000× *g*, 1 h		TEM, WB	CD63, CD81, TSG101	NR	miR-146a-5p
Wang [24]	nasal mucus	centrifugation (3000× *g*, 15 min) ExoQuick precipitation kit (30 min, 4 °C) [49] centrifugation (1500× *g*, 30 min, 4°C), removing of supernatant via aspiration, centrifugation (1500× *g*, 5 min, 4 °C)		TEM, WB	CD9, CD63	NR	LncRNA NEAT1
Samra [25,31]	urine	centrifugation (10,000× *g*, 10 min, 4 °C) [50], filtration using 0.22 µm filter		NR	NR	NR	bacterial DNA
Mo [26]	BMDCs, T cells	differential ultracentrifugation [51]		TEM, WB	CD9, CD63, CD81, MHC-II	NR	OVA/MHC-II, FLLL31
Mariani [27]	serum	differential ultracentrifugation: 1000× *g*, 15 min, 4 °C; 2000× *g*, 15 min, 4 °C; 3000× *g*, 15 min, 4 °C; resuspending in PBS and filtration 0.1 µm pore-size polyethersulfone filter; 110,000× *g*, 94 min, 4 °C [46]		TEM, NTA, FC	NR	NR	NR
Fang [28]	serum, nasal mucus	differential ultracentrifugation: 2000× *g*, 20 min, 4°C; 12,000× *g*, 30 min, 4 °C; 110,000× *g*, 2 h, 4 °C; 110,000× *g*, 2 h and 70 min, 4°C [47]		ELISA, NTA, TEM, WB, FC	CD9, CD63, CD81, TSG101, Alix	NR	Derp 1
Zhu [29]	RPMI 2650	differential ultracentrifugation: 12,000× *g*, 45 min, 4 °C; dilution in PBS; 110,000× *g*, 2 h, 4 °C; resuspending in PBS and filtration using 0.22 µm filter; 110,000× *g*, 70 min, 4 °C [47]		TEM, WB	CD63, CD81	calnexin	LncGAS5
Fang [30]	iPSCs-derived MSCs [52], fibroblasts	anion-exchange chromatography [53]	differential ultracentrifugation: 300× *g*, 5 min; 2000× *g*, 20 min; 12,000× *g*, 30 min, 4 °C; 110,000× *g*, 2 h, 4 °C; washed with ice-cold PBS; 110,000× *g*, 70 min, 4 °C [47]	FC, NTA, TEM, WB	CD9, CD63, CD81, Alix and TSG101	calnexin	miR-146a-5p
Wu [32]	nasal mucus	gradient ultracentrifugation: 3000× *g*, 15 min; 10,000× *g*, 30 min; 100,000× *g*, 60 min [54]		FACS	CD63, MHC-II	NR	various miRNAs
Luo [33]	RPMI 2650, A549	refer to Qiu, 2011		qPCR, WB	LMP1 protein	NR	miR-146a
Qiu [34]	nasal mucosa, RPMI 2650	gradient centrifugation: 300× *g*, 10 min; 1200× *g*, 20 min; 10,000× *g*, 30 min [55]		TEM, FC, WB	NR	NR	Derp 1, SEB

A549—mouse airway epithelial cell line; BMDC—bone-marrow-derived dendritic cell; CD—cluster of differentiation; DLS—dynamic light-scattering*;* ELISA—enzyme-linked immunosorbent assay; FACS—fluorescence-activated cell sorting; FC—flow cytometry; FLLL31—tetramethylcurcumin (a curcumin analog); HEK293—human embryonic kidney 293; HSP70—heat shock protein 70; iPSCs—induced pluripotent stem cells; LncGAS5—long-noncoding RNA Growth Arrest Specific 5; LncRNA—long non-coding RNA; MHC—major histocompatibility complex; miR = miRNA—micro ribonucleic acid; MSCs—mesenchymal stromal cells; NEAT1—Nuclear Paraspeckle Assembly Transcript 1; NR—not reported; NTA—nanoparticle tracking analysis; OVA—ovalbumin; PBS—phosphate-buffered saline; qPCR—quantitative polymerase chain reaction; RPMI 2650—human airway epithelial cell line; SEB—Staphylococcal enterotoxin B; TEM—transmission electron microscopy; Tfhs—follicular helper T cells; TSG101—tumor susceptibility gene 101; WB—Western blotting.

## Data Availability

Not applicable.

## Supplementary Materials

The following supporting information can be downloaded at: https://www.mdpi.com/article/10.3390/ijms24010367/s1.

## Abbreviations

A549	mouse airway epithelial cell line
AR	allergic rhinitis
BMDC	bone-marrow-derived dendritic cell
CD	cluster of differentiation
CRS	chronic rhinosinusitis
DC	dendritic cell
Derp	*Dermatophagoides pteronyssinus*
Derp (1)	*Dermatophagoides pteronyssinus* (protease)
ELISA	enzyme-linked immunosorbent assay
EZH2	enhancer of zeste homolog 2
FC	flow cytometry
FLLL31	tetramethylcurcumin (a curcumin analog)
GATA-3	GATA binding protein-3
GM-CSF	granulocyte–macrophage colony-stimulating factor
HDM	house dust mite
HLA-DR	Human Leukocyte Antigen—DR isotype
HNECs	human nasal epithelial cells
HUC-MSC-EVs	human umbilical cord mesenchymal stem cell-derived extracellular vesicle
HS	healthy subject
iDC	immature dendritic cell
IFN-ɣ	interferon ɣ
iPSC	induced pluripotent stem cells
KEGG	Kyoto Encyclopedia of Genes and Genomes
LncGAS5	long non-coding ribonucleic acid Growth Arrest Specific 5
lncRNA	long non-coding ribonucleic acid
mDCs	mature dendritic cell
MHC	major histocompatibility complex
miR = miRNA	micro ribonucleic acid
mRNA	messenger ribonucleic acid
MSC	mesenchymal stromal cell
NEAT1	Nuclear Paraspeckle Assembly Transcript 1
NR	not reported
NR4A2	Nuclear Receptor Subfamily 4 Group A Member 2
ov	overexpressed vectors
OVA	ovalbumin
PBS	phosphate-buffered saline
PM	particulate matter
RPMI 2650	human airway epithelial cell line
SCIT	subcutaneous immunotherapy
SEB	Staphylococcal enterotoxin B
EV	extracellular vesicle
TEM	transmission electron microscopy
Tfhs	follicular helper T cells
Treg	regulatory T cell
WB	Western blotting
