# Supplementary material for: Application of Extracellular Vesicles in Allergic Rhinitis: A Systematic Review
Source: Int J Mol Sci. 2022 Dec 26;24(1):367. doi: 10.3390/ijms24010367 (PMC9820222; doi:10.3390/ijms24010367)
Supplement: Supplementary file 1 [file ijms-24-00367-s001.zip › ijms-2101993-supplementary.pdf]

Table S1. The search strategy for each database.

**Pubmed**
(("Exosomes"[Mesh]) OR (exosome) OR (extracellular vesicle*) OR (exosomal) OR (liquid biopsy)) AND ("Rhinitis, Allergic"[Mesh])
**Scopus**
TITLE-ABS-KEY ((exosome*) OR (extracellular vesicle*) OR (exosomal)) AND (allergic rhinitis)
**Cochrane**
#1 "Exosomes"[Mesh]<br>#2 (exosome) OR (extracellular vesicle*) OR (exosomal) OR (liquid biopsy)<br>#3 "Rhinitis, Allergic"[Mesh]<br>(#1 OR #2) AND #3
**Web of Science**
(AB=((exosome*) OR (extracellular vesicle*) OR (exosomal) OR (liquid biopsy))) AND (AB=allergic rhinitis)

Table S2. General characteristics of the included studies.

Study	Year	Country	Type of Study	Aim	Main Steps of Study Procedure	Main Findings
Teng [17]	2022	China	*in vitro* and *in vivo*, animal study	To explore the relationship between Tfhs and DC maturation in AR.	● Tfhs were isolated from OVA-sensitized mice and co-cultured with DCs derived from mouse bone marrow.<br>● Tfhs exosomes were extracted and exosomal miRNAs were analyzed.<br>● DCs treated with miR-142-5p mimics or inhibitors or transfected with CDK5 small interfering RNAs.<br>● The exosomes of AR-derived Tfhs were injected intravenously into healthy mice.	● AR-derived Tfh exosomes contributed to DS maturation and promoted AR in mice.<br>● MiR-142-5p was differentially decreased in AR-derived exosomes.<br>● CDK5 was predicted to be the target gene for the direct action of miR-142-5p.<br>● MiR-142-5p inhibited DC maturation by inhibiting CDK5 expression.<br>● Inhibition of the STAT3 signaling pathway can reverse the regulation of miR-142-5p/CDK5 on DC maturation
Peng [18]	2022	China	*in vitro*	To evaluate the effects of MSC-EV on DCs in AR.	● EV isolated from the iPSC-MSCs.<br>● Human monocyte-derived DCs generated and cultured with MSC-EV to differentiate EV-iDCs and EV-mDCs.<br>● EV-mDCs co-cultured with isolated CD4+ T cells or PBMCs from AR patients<br>● Examination of the levels of Th1 and Th2 cytokines produced by T cells.	● MSC-EV inhibited the differentiation of human monocytes to iDCs with downregulation of CD40, CD80, CD86, CD11c, and HLA-DR expression.<br>● EV-mDCs suppressed the Th2 immune response by reducing the production of IL-4, IL-9, and IL-13 via IL-10.<br>● EV-mDCs promoted the expansion of Treg cells.
Liu [19]	2022	Japan	*in vitro* and *in vivo*, animal study	To evaluate EVs as delivers of allergen (OVA) and CpG DNA for treatment of AR.	● Preparing EVs loaded with CpG DNA and OVA.<br>● The uptake of the CpG-OVA-EVs by DCs and their activation were evaluated *in vitro*.<br>● The CpG-OVA-EVs were administered intranasally to the mice.<br>● The therapeutic effect of the CpG-OVA-EVs on allergic symptoms and the immune system was evaluated on an AR mouse model.	● The CpG-OVA-EVs with the size of 90 nm and average zeta potential of -30 mV were obtained and could activate DCs.<br>● The CpG-OVA-EVs delivered to the NALT significantly enhanced OVA-specific IgG antibody titers, decreased the level of serum IgE and alleviated allergic symptoms compared to the control group in mice models of AR.

Li [20]	2022	China	*in vitro*	To determine the role of Lnc00632 in the development of AR.	• Isolation of CD4+ T cells from peripheral blood of AR patients.<br>• CD4+ T cells incubated with anti-CD3, anti-CD28, IL-4, IL-2, anti-IFN-γ, and the control-EVs or HUC-MSC-EVs for 7 days.<br>• MSC cells transfected with si-Lnc00632 or si-NC and isolation of si-Lnc0063-EVs and si-NC-EVs.<br>• CD4+ T cells transfected with si-Lnc0063-EVs and si-EZH2 and with ov-Lnc00632 or ov-Lnc00632 and ov-GATA-3 before induction of Th2 differentiation.<br>• Total RNA extracted from human AR tissues, CD4+ T cells, and MSC-EVs.<br>• Detection of the levels of IL-4 and IL-13 in cell supernatants.	• The expression of Lnc00632 was significantly decreased in the nasal mucosa of AR patients.<br>• MSC-derived exosomes inhibited Th2 differentiation and decreased GATA-3 expressions and IL-4 levels in CD4+ T cells.<br>• Knockdown Lnc00632 partially reversed the effects of exosomes on Th2 differentiation, IL-4, and IL-13 levels, and GATA-3 expression.<br>• Lnc00632 overexpression suppressed Th2 differentiation of CD4+ T cells, reduced IL-4 and IL-13 levels, and GATA-3 expressions.<br>• Lnc00632 suppressed the expression of GATA-3 by interacting with EZH2.<br>• GATA-3 overexpression partially reversed the effect of Lnc00632 on the Th2 differentiation of CD4+ T cells.
Jiang [21]	2022	China	cross-sectional study	To determine the profile of miRNAs in serum exosomes of AR children and evaluate their capacity for predicting SCIT response.	• There was high-throughput sequencing used to identify the miRNA of serum exosomes in pediatric AR.<br>• Evaluation of the ability of differentially expressed miRNAs in predicting the efficiency of SCIT in AR children.	• Among 812 miRNAs detected in the serum exosomes, 16 were upregulated and 14 downregulated.<br>• Hsa-miR-4669 was significantly downregulated in the effective group than the ineffective group.<br>• Hsa-miR-4669 level was correlated with the VAS and TNSS.<br>• Hsa-miR-4669 level presented a reliable accuracy in predicting SCIT efficiency in AR children (AUC=0.785).
Chiang [22]	2022	China	cross-sectional study	To compare the diversity in EVs and the microbiome composition between AR patients and HS.	• Levels of eosinophils and serum IgE were measured in AR patients and HS.<br>• Nasal EVs were identified and 16S rRNA sequencing was used to profile the microbial communities.<br>• Microbial diversity was determined using alpha and beta diversities.	• In AR patients eosinophils, total serum IgE, and sIgE to *Dermatophagoides* were elevated.<br>• Alpha diversity in nasal EVs from AR patients was lower compared to the HS group.<br>• There were microbiome differences between the AR and HS groups demonstrated in beta diversity.

					● Microbial metabolic pathways characterized using PICRUst2 and KEGG analyses.	● Compared to HS, in the AR group significantly higher levels of the genera *Acetobacter*, *Mycoplasma*, *Escherichia*, and *Halomonas* and significantly lower levels of *Zoogloea*, *Streptococcus*, *Burkholderia*, and *Pseudomonas* were observed.<br>● From 35 microbial metabolic pathways recognized in AR and HS groups, 25 pathways were more abundant in the AR group.
Zhou [23]	2021	China	*in vitro*	To investigate the role of the mi-R-146a-5p/SERPINB2 signaling pathway in the differentiation of Th1 and Th2 cells in AR.	● Collection of blood samples from AR patients and HS.<br>● The population of Th1 and Th2 cells were characterized using flow cytometry.<br>● Isolation of exosomes from MSC cultures and coculture of CD4+ T cells with PKG-67-labeled MSC-EVs.<br>● Detection of the expression of GATA-3, SERPINB2, and population of Th2 cells after coculture of CD4+ T cells with MSC-EVs, GW4869-MSC-EVs, and upregulated and downregulated in miR-146a-5p MSC-EVs.<br>● Investigation of the correlation between miR-146a-5p and SERPINB2, and the role of SERPINB2 in the differentiation of Th2 cells.	● The population of Th2 cells was significantly elevated in AR patients compared to HS.<br>● MSC-EVs could decrease the expression of SERPINB2 and the differentiation of Th2 cells.<br>● Exosomal miR-146a-5p exhibited consistent effects and lowered the expression of SERPINB2 by binding on its 3'UTR.<br>● The differentiation of Th2 cells was promoted by SERPINB2 which could be reversed by MSC-EVs.<br>● MiR-146a-5p expression was negatively associated with the SERPIN B2 expression in the serum of AR patients.
Wang [24]	2021	China	*in vitro*	To explore the function and molecular mechanism of LncRNA NEAT1 in AR.	● Collection of nasal mucosal samples from inferior turbinate mucosa from AR patients and HS.<br>● Exploration of NEAT1 levels in the nasal mucosa and exosomes from AR patients and HS.<br>● IL-13-treated primary HNECs culture was	● NEAT1 levels were upregulated in the nasal mucosa and mucus-derived exosomes in AR and IL-13-treated HNECs.<br>● NEAT1 knockdown significantly suppressed levels of GM-CSF, eotaxin-1, and MUC5AC and apoptosis rate, but promoted the viability of IL-13-treated HNECs.

					used to establish the AR model.<br>● Detection of the mRNA levels of NEAT1, miR-511, and NR4A2 by RT-qPCR.<br>● Examination of the protein levels of exosomal markers by WB.<br>● Determination of the levels of GM-CSF, eotaxin-1, and MUC5AC by ELISA.<br>● Evaluation of the cell viability and apoptosis by CCK-8 and TUNEL assays.<br>● Extraction of exosomes from the cell culture medium.	● Exosomes containing NEAT1 induced inflammatory cytokine production and apoptosis, while NEAT1 depletion inhibited these effects.<br>● NEAT1 directly interacted with miR-511, which could bind to the 3′UTR of NR4A2.<br>● The inhibition of miR-511 promoted inflammatory cytokines, mucus production, and apoptosis in IL-13-induced HNECs, which was counteracted by depleting NR4A2.
Samra [25]	2021	Korea	cross-sectional study	To explore the composition and function of bacteria-derived EVs genes in urine to look for characteristics of AR.	● Nucleic acid extraction from urinal EVs<br>● 16S rRNA sequencing.<br>● Characterization using α-diversity, β-diversity, network analysis, intergroup comparison of bacterial composition and predicted functions, and correlation with total IgE, percentage of eosinophils, and fractional exhaled nitric oxide.	● The compositional α-diversity was the highest while functional α-diversity was the lowest in the allergic airway group.<br>● The allergic airway group featured the least intersample variation.<br>● Klebsiella, Haemophilus, members from Lachnospiraceae and Ruminococcaceae, and the pathways of sphingolipid and glycerolipid metabolism, and biosynthesis of peptidoglycan and lysine were the highest in the allergic airway group and positively correlated with total IgE or percentage of eosinophils.<br>● Genetic information processing function was the highest in the allergic airway group and contributed to 48% of the intergroup variance.
Mo [26]	2021	China	*in vitro* and *in vivo*, animal study	To induce antigen-specific Tregs through the use of EVs that carry two types of T cell activators -	● Obtaining BMDCs and T cells from naive mice.<br>● Preparing 4 types of BMDCs-derived EVs: OFexo (containing OVA and FLLL31), Oexo (containing OVA), Fexo (containing FLLL31), and Eexo (containing neither OVA nor FLLL31), and T cells-derived-EVs	● Administration of OFexo-containing nasal instillation increased IL-10+ CD25+ T cells (Tr1 cells) in the mouse airway tissues, while administration of Oexo, Eexo, or Fexo did not show such an effect, and it was also confirmed in in-vitro experiments.<br>● Analysis of naive CD4+ T cells isolated from

				OVA/MHC-II and FLLL31 (an analog of curcumin).	OFexo (control EVs).<br>● OVA-specific CD4+ T cells were isolated from the mouse spleen and cultured with EVs labeled with FITC.<br>● Mice were treated with OFexo or control EVs in nasal drops and then Tr1 cells from their nasal mucosa and lungs were assessed.<br>● A murine model of AR was developed with OVA.<br>● AR mice were treated with nasal instillation containing OFexo or control EVs	the mouse spleen, cultured in the presence of OFexo revealed an increase in the expression of c-Maf, the *Il10* gene transcription factor, in CD4+ T cells.<br>● *In vitro*-produced Tr1 cells induced by OFexo effectively suppressed the proliferation of CD4+CD25- effector T cells.<br>● Treatment of mice with induced AR with OFexo resulted in effective suppression of the AR response, which was abolished by the presence of the IL-10 inhibitor.<br>● The significantly reduced number of Tr1 cells in AR-induced mice compared to naive control mice was increased by OFexo administration (which was abolished by the IL-10 inhibitor).
Mariani [27]	2021	Italy	cross-sectional study	To investigate the effects of PM10 and PM2.5 exposure on bacterial and host-derived EVs and the nasal microbiome.	● Subjects were assigned the daily PM10 and PM2.5 concentration from the place of residence within 6 days preceding sampling.<br>● DNA was extracted from nasal swabs and 16S rRNA gene sequencing was conducted.<br>● Exosomes were isolated from blood samples and analyzed.	● A significantly higher median concentration of plasmatic EVs in AR compared to HS.<br>● PM exposure had a different impact on EV release and nasal microbiome composition in AR and HS groups.
Fang [28]	2021	China	*in vitro*	To verify the presence of Derp 1 and antigen presentation-related molecules on plasma-derived EVs, to investigate the association of Derp 1-carrying	● Plasma-derived EVs from HS and AR patients were isolated and characterized.<br>● Expression of Derp 1 and antigen-presenting molecules on EVs was determined by WB, FC, and ELISA.<br>● It was investigated whether there was an association between the levels of Derp 1 on EVs derived from plasma and nasal secretion with the severity of AR.<br>● The effect of plasma-derived EVs on Th2	● There were no significant differences in the concentration of particles and expression for specific EV markers between HS and AR patients, and both also had a structural lipid layer.<br>● Derp 1 levels on plasma-derived EVs from AR patients were significantly higher than those from HS and were also significantly higher on plasma-derived EVs from patients with mild

				EVs with AR severity, and to examine the effect of plasma-derived EVs from AR patients on Th2 cell proliferation and differentiation.	cell development and CD4+ T-cell proliferation was investigated.	AR compared to those with moderate-severe AR.<br>• There were no significant differences in the levels of antigen-presenting molecules on plasma-derived EVs between HS, mild AR, and moderate-severe AR patients.<br>• Derp 1 levels on plasma EVs were significantly associated with AR patients' symptom score (VAS and TNSS), but no such correlation was observed for Derp 1 levels in nasal secretions.<br>• Plasma-derived EVs from AR patients significantly increased the levels of Th2 cells and IL-13 and, conversely, decreased the levels of Th1 and Th17 cells, while no effect on CD4+ T-cell proliferation was observed.
Zhu [29]	2020	China	*in vitro*	To investigate whether, under AR conditions, exosomes derived from nasal epithelium encapsulate LncGAS5 and transport it to CD4+ T cells, promoting allergic reactions by suppressing Th1 differentiation and increasing Th2 differentiation.	• The different expressions of LncGAS5 in nasal epithelial samples were compared between AR patients and HS and between human epithelial cells RPMI 2650 with or without OVA simulation.<br>• The expression of LncGAS5 was determined in the exosomes isolated from nasal mucus samples of AR patients and HS and human epithelial cells (RPMI 2650) with or without OCA.<br>• Th1/Th2 differentiation was induced in naive CD4+ T cells, and the exosomes isolated from nasal mucus samples of AR patients and HS were added for the 7-day coincubation, and the percentage of IFN-γ expressing cells (Th1 cells) and IL-4 expressing cells (Th2 cells) were detected using FC, WB analysis, and ELISA.	• LncGAS5 was upregulated in AR epithelial samples, exosomes isolated from nasal mucus samples of AR patients, and OVA-induced RPMI 2650 cells.<br>• The coincubation of exosomes isolated from nasal mucus samples of AR patients and CD4+ T cells suppressed Th1 differentiation and promoted Th2 differentiation.<br>• Coincubation with exosomes isolated from OVA-induced RPMI 2650 cells reduced the mRNA level and the protein level of T-bet and inhibited transcription and expression of EZH2, and coincubation with exosomes isolated from OVA-induced RPMI 2650 cells but after downregulation off LncGAS5 negated such response.<br>• The gain-of-function and loss-of-function experiments suggested that LncGAS5 in exosomes isolated from OVA-induced RPMI

						2650 cells mediates Th1/Th2 differentiation partly through downregulation T-bet and EZH2.
Fang [30]	2020	China	*in vitro* and *in vivo*, animal study	To evaluate an anionic-exchange chromatography protocol for MSC-EV isolation, to evaluate the relationship of MSC-EV to human ILC2 function and the effect on allergic airway inflammation in mice, and to evaluate the effect of miR-146a-5p in MSC-EV on allergic airway inflammation in mice.	• Comparison of anionic chromatography and differential ultracentrifugation for MSC-EV isolation.<br>• To assess the effect of MSC-EV on ILC2 function from AR patients and in mouse ILC2-dominant asthma model.<br>• Evaluation of MSC-EV uptake by ILC2, using mCherry-labeled MSC-EVs administered both *in vitro* and systemically *in vivo*.<br>• Total small RNA sequencing of MSC-EV and Fb-EV, which contributed to the therapeutic effect of MSC-EV on allergic airway inflammation.<br>• Determining precisely whether miR-146a-5p was involved in the effect of MSC-EV on ILC2-dominated airway inflammation.	• Anion-exchange chromatography showed superiority over differential ultracentrifugation in terms of obtaining more EVs, shorter isolation time, lower cost, higher reproducibility, and no damage for EVs.<br>• MSC-EV but not Fb-EV were found to significantly inhibit the function of human ILC2 - MSC-EV significantly reversed the high levels of IL-9 and IL-13 in the supernatants of sorted ILC2s from the human buffy coat in response to IL-2/25/33.<br>• Systemic administration of MSC-EV but not Fb-EV exhibited inhibition of ILC2 levels, infiltration of inflammatory cells in the peritracheal area, and numbers of epithelial goblet cells in mouse lung tissues, decreased numbers of total inflammatory cells, eosinophils, and neutrophils, levels of IL-5 and IL-13 in BALF, reduced the airway hyperresponsiveness in ILC2- dominant allergic airway inflammation.<br>• Using RNA sequencing, miR-146a-5p was identified as one of the most significant upregulated miRNAs in MSC-EV and selected as the candidate to mediate the above effects of MSC-EV.<br>• It was demonstrated *in vitro* and in a mouse model that the transfer of miR-146a-5p in MSC-EV to ILC2 in part contributed to the effects of MSC-EV on ILC2.

Samra [31]	2019	Korea	cross-sectional study	To evaluate urine EVs and determine their utility as biomarkers for monitoring allergic airway diseases in children.	• Collection of urine samples and isolation of EVs.<br>• DNA extraction from EVs	• AR children had a significantly higher Chao-1 richness index than HS.<br>• Principal component analysis revealed dysbiosis in CRS, AR, and atopic asthma compared to the HS.<br>• One phylum and 19 families and genera were significantly enriched or depleted in the CRS, AT, and atopic asthma groups compared to HS.
Wu [32]	2015	China	cross-sectional study	To characterize the miRNA contained in exosomes in AR.	• Exosomes were isolated from the nasal mucus of AR patients and HS.<br>• Exosomal RNA was analyzed and selected findings were validated with quantitative RT-PCR.<br>• Bioinformatic analysis by DIANA-mirPath.	• Compared to HS, in the nasal mucus of AR patients, 21 exosomal miRNAs were significantly upregulated (let-7a, miR-454, let-7b, miR-223, miR-483-5p, miR-149*, miR-30a-5p, miR-193b, miR-199b-3p, miR-210, miR-184, miR-190, miR-199b, miR-203, miR-219, miR-299-5p, miR-302b, miR-335, miR-518f, miR-627, miR-708), and 14 were downregulated (miR-874, miR-208, miR-875-5p, miR-302c*, miR-146a, miR-155, miR-122, miR-181a, miR-28-3p, miR-885-5p, miR-24, miR-136, miR-548d, miR-628-5p).<br>• There were 32 KEGG biological processes found to be significantly enriched including the B-cell receptor signaling pathway, the natural killer cell-mediated cytotoxicity, the T-cell receptor signaling pathway, the RIG-I-like receptor signaling pathway, the Wnt signaling pathway, endocytosis, and salivary secretion.
Luo [33]	2015	China	*in vitro* and *in vivo*, animal study	To test whether miR-146a induces the expression of IL-10 in monocytes.	• The levels of miR-146a were determined by real-time RT-PCR in human nasal epithelial cells of AR patients, HS, and RPMI2650 cells after exposure to LPS and Th2 cytokines (IL-4, IL-5, IL-13).<br>• The miR-146a-laden exosomes were	• The levels of miR-146a expressed by nasal epithelial cells were markedly lower in the nasal epithelial cells of AR patients compared to HS.<br>• miR-146a levels were significantly increased in RPMI2650 culture after exposure to LPS, in

					generated with nasal epithelial cell line, RPMI2650 cells.<br>● CD14+ CD16- monocytes were isolated from PBMCs of HS and cultured in the presence of the miR-146a-laden exosomes at gradient doses for 6 days.<br>● The IL10+ monocytes were evaluated by flow cytometry.<br>● A mouse AR model with the OVA as a specific antigen was developed and AR mice were transferred with conditioned monocytes (exposed to miR-146a-laden exosomes in the culture) or nasal drops containing the miR-146a-laden exosomes.<br>● Groups of AR mice and naive mice were treated with saline or nasal drops containing the miR-146a-laden exosomes and miR-146-null exosomes.	a LPS dose-dependent manner, whereas exposure to Th2 cytokines significantly reduced the levels of miR-146a in the nasal epithelial cells.<br>● Exposure to miR-146a-laden exosomes increased the expression of IL-10 in the monocytes at both mRNA and protein levels in a miR-exosomes dose-dependent manner and also increased the IL-10 promoter activity and the IL-10 promoter methylation.<br>● An OVA-specific spleen Teff cell proliferation was induced in the culture from AR mice, which was significantly suppressed in mice that received the conditioned monocytes or nasal drops containing the miR-146a-laden exosomes.<br>● The treatment with miR-146a-laden exosomes increased the frequency of IL-10+ monocytes in the nasal mucosa in both naive mice and AR mice, which did not occur when the mice were treated with the nasal drops containing the miR-146a-null exosomes.
Qiu [34]	2011	China	*in vitro*	To explore the role of antigen-specific CD8+ T cells in the pathogenesis of chronic atypical AR.	● Exosomes were purified from the nasal epithelial cell line, RPMI cells, and nasal mucosal epithelial samples obtained from patients with chronic obstruction atypical AR.<br>● Exosomes were examined by immune gold electron microscopy for SEB and Derp1.<br>● Isolation of CD3+ CD25- T cells from human PBMCs.<br>● On cell culture models, it was observed how exosomes affect modulating DC's properties and how exosome-stimulated DCs influence naive T cell differentiation	● Exosomes obtained from chronic atypical AR patients carried microbial products, SEB, and airborne antigen, Derp1.<br>● Stimulation of DCs with exosomes carrying SEB/Derp1 significantly increased levels of CD80, CD86, and MHC-I in DC.<br>● DCs stimulated by exosomes could induce the differentiation of CD3+ T cells into CD8+ T cells.<br>● Exposure to the specific antigen resulted in the release of granzyme B and perforin by CD8+ T cells and more than 30% of antigen-specific CD8+ T cells proliferated.

					and antigen-specific CD8+ T cell activation.	

3′UTR - 3′ untranslated region; AR - allergic rhinitis; BALF - bronchoalveolar lavage fluid**;** BMDC - bone marrow-derived dendritic cell; CCK-8 - Cell Counting Kit-**8;** CD - cluster of differentiation; DC - dendritic cell; Derp (1) - *Dermatophagoides pteronyssinus* (protease); ELISA - enzyme-linked immunosorbent assay; EV – extracellular vesicle; EZH2 - enhancer of zeste homolog 2; Fb – fibroblast; FC – flow cytometry; FITC - fluorescein isothiocyanate; FLLL31 - tetramethylcurcumin (a curcumin analog); GATA-3 - GATA binding protein-3; GM-CSF - granulocyte-macrophage colony-stimulating factor; HLA-DR - Human Leukocyte Antigen – DR isotype; HNECs - human nasal epithelial cells; HUC-MSC-EVs - human umbilical cord mesenchymal stem cell-derived extracellular vesicles; HS - healthy subject; iDC - immature dendritic cell; IFN-ɣ - interferon ɣ; ILC2 - group 2 innate lymphoid cells; iPSC - induced pluripotent stem cells; KEGG - Kyoto Encyclopedia of Genes and Genomes; Lnc00632 - long intergenic non-protein coding RNA 632; LncGAS5 - long non-coding RNA Growth Arrest Specific 5; LPS – lipopolysaccharide; mDC - mature dendritic cell; MHC - major histocompatibility complex; miR = miRNA - micro ribonucleic acid; MSC - mesenchymal stromal cell; NALT - nasopharynx-associated lymphoid tissue; NC - negative control; NEAT1 - Nuclear Paraspeckle Assembly Transcript 1; NR4A2 - Nuclear Receptor Subfamily 4 Group A Member 2; ov - overexpressed vectors; OVA – ovalbumin; PBMCs - peripheral blood mononuclear cells; PICRUst2 - Phylogenetic Investigation of Communities by Reconstruction of Unobserved States; PM - particulate matter; rRNA - ribosomal ribonucleic acid; RT-PCR - reverse-transcription polymerase chain reaction; RT-qPCR - quantitative reverse-transcription polymerase chain reaction; SCIT - subcutaneous immunotherapy; SEB - Staphylococcal enterotoxin B; si - small interfering; Teff - effector T lymphocyte; Tfhs - Tfhs - follicular helper T cells; TNSS – total nasal symptom score; Tr1 - antigen-specific type 1 regulatory T cell; Treg - regulatory T cell; VAS – visual analog scale; WB - western blotting

Table S3. Participants in human studies with inclusion and exclusion criteria of the study group.

Study	Study group	Control group	Inclusion Criteria for study groups	Exclusion criteria for study groups
Peng [18]	AR patients (n = 5)	„anonymous donors" for human blood buffy coats (n = 47)	● the nasal and/or ocular hypersensitivity symptoms and sIgE tests	● pregnant,<br>● current smokers or ex-smokers with more than 10 pack years,<br>● oral or nasal corticosteroid or other treatments (e.g. H1-antihistamine or immunotherapy) used for 6 weeks before the study
Li [20]	AR patients (n = 30)	HS (n = 30)	● a history of >2 years of typical AR, with clinical manifestations of runny nose, positive allergen SPT, and sIgE level > 0.3 IU/mL.<br>● not received either topical or systemic corticosteroid treatment for 4 weeks before the recruitment	● subjects with sinusitis
Jiang [21]	AR children (n = 8), including the effective group (n = 4) and the ineffective group (n = 4)<br>the first validation group (n = 52),<br>the second validation group (n = 50)	-	● aged 6-14 years old<br>● receiving SCIT for more than 1 year<br>● diagnosed with HDM-induced AR, referring to the ARIA guidelines [3]<br>● positive results of SPT to Derf and/or Derp (at least ++) and/or s-IgE level against Derf or Derp (>0.35 IU/mL)<br>● informed consent provided by all children's guardians [47]	● acute exacerbation in asthma<br>● vasomotor rhinitis and other nasal or sinus diseases<br>● a history of immunotherapy<br>● consumption of anti-allergic drugs within 4 weeks before enrollment [47]

Chiang [22]	AR patients (n = 20)	HS (n = 19)	● aged 15-36 years old<br>● AR diagnosed according to the ARIA guidelines [3] patients with a symptom complex that consists of any combination of congestion, rhinorrhea, sneezing, nasal itching, and symptoms were present more than 4 days/week and for more than 4 consecutive weeks<br>● a history of typical rhinitis symptoms relevant to exposure to mites<br>● mite-sensitized AR patients diagnosed by *in vitro* testing for allergen panels of Derp, Derf, and 16 (*Blattella germanica*)<br>● written informed consent	● AR patients with a seasonal onset tendency<br>● symptoms of CRS, nasal polyposis, immunological disease, neurodevelopmental disabilities, or respiratory infections.<br>● use of probiotics, systemic antibiotics, or steroids within 14 days before the study
Zhou [23]	AR patients (n = 20)	HS (n = 20)	● age 20-50 years<br>● daytime fatigue, daytime somnolence, nasal congestion<br>● perennial AR with a positive SPT response for the perennial allergen (wheal diameter equal to 3 mm or greater)<br>● a negative SPT response for seasonal allergens	● any other chronic medical conditions or allergic disorders except AR, seasonal allergies, known sleep apnea, obesity, nasal polyps, recent upper respiratory tract infection, deviated septum, asthma, and other respiratory diseases
Wang [24]	AR patients (n = 30)	HS (n = 30)	● perennial AR<br>● written informed consent	● receiving topical or systemic corticosteroid therapy before the first 4 weeks of enrollment
Samra [25]	the allergic airway group (n = 16), atopic controls (n = 7)	HS (n = 26)	● rhinitis was considered present if there were rhinitis symptoms and rhinitis treatment<br>● asthma was considered present if there were asthma symptoms and asthma treatment<br>● sensitization to aeroallergens was diagnosed by a positive SPT or positive sIgE<br>● an average BMI<br>● normal urine analysis<br>● the absence of current respiratory illness	NR

Mariani [27]	AR patients (n = 26)	HS (n = 24)	● AR was diagnosed according to the ARIA guidelines [3]<br>● a positive standard battery of the SPT for at least one inhalant allergen and/or eosinophil count<br>● written informed consent	● diabetes, hypertension, autoimmune diseases, cancer, or other major chronic health conditions (e.g. chronic and/or allergic asthma)<br>● pregnancy<br>● history of illicit drug use
Fang [28]	AR patients (n = 18)	HS (n = 9) and „anonymous donors" for human blood buffy coats (number NR)	● AR was diagnosed according to ARIA criteria, including a history of nasal symptoms (rhinorrhea, nasal itching, nasal obstruction, sneezing, and postnasal drip)<br>● patients positive for Derp 1 determined by a positive SPT for HDM and sIgE tests (>0.35 IU /mL)<br>● written informed consent	● use of antihistamines, topical or systemic steroids, and biologics within 1 month before the study<br>● history of any other diseases or smoking<br>● differences in occupational exposure to aeroallergens or residential areas between HS, and subgroups of AR (mild AR and moderate-severe AR)
Zhu [29]	AR patients (n = 30)	HS (n = 30)	refer to Luo 2015	refer to Luo 2015
Fang [30]	AR patients (n = 40)	„anonymous donors" for human blood buffy coats (n = 12)	● the diagnosis of AR following the criteria of the initiative from ARIA 2008 guidelines [48]<br>● positive SPT result for Derp/Derf and sIgE tests to Derp/Derf<br>● age 20-61 years<br>● written informed consent	● use of antihistamines or intranasal steroids within 1 month before the study, or oral steroids within 3 months before the study
Samra [31]	AR patients (n = 39), CRS patients (n = 27), atopic asthma patients (n = 19)	HS (n = 33)	● age 5-12 years<br>● average BMI<br>● no concurrent major health concern<br>● written informed consent from the patients and their parents/guardians<br>➔ CRS patients: nasal symptoms for more than 12 weeks and negative SPT<br>➔ atopic patients: sensitivity to one or more	● a history of antibiotic intake within 2 weeks before urine sampling<br>● presence of urinary symptoms

			aeroallergens via SPT and/or sIgE<br>➔ AR patients: the presence of one or more nasal symptoms without nasal polyps<br>➔ atopic asthma patients: at least one asthma attack in the previous 12 months with PC_{20} of 16 mg/mL or less	
Wu [32]	AR patients (n = 54)	HS (n = 30)	● the diagnosis of AR based on the patient's medical history, symptoms, and the presence of a positive SPT in response to a panel of common allergens defined by the ARIA 2008 guidelines [48]<br>● airway responsiveness to methacholine with FEV_1 higher than 70% of predicted<br>● written informed consent was obtained from patients and their parents in the case of children	● bronchial asthma, CRS, nasal polyposis, excessive septal deviation<br>● current smoking<br>● using anti-histamines for 10 days before testing
Luo [33]	AR patients (n = 10)	HS (n = 10)	• the diagnosis of AR was based on a typical history of AR of more than 2 years, with the clinical manifestation including a running nose, positive SPT against specific antigens, and serum sIgE levels greater than 0.3 IU/mL	• sinusitis
Qiu [34]	atypical AR patients (n = 12).	patients with nasal cancer (the nasal mucosa collected from the marginal non-cancer tissue) (number NR)	● patients with atypical AR – nasal obstruction, inferior turbinate hypertrophy, positive SPT to one to several airborne antigens (obligatory sensitization to Derp 1), but no typical symptoms of AR.	NR

AR – allergic rhinitis; ARIA - Allergic Rhinitis and its Impact on Asthma; BMI - Body Mass Index; CRS – chronic rhinosinusitis; Derf - *Dermatophagoides farina*; Derp (1) - *Dermatophagoides pteronyssinus* (protease); FEV_1 - *forced expiratory volume in 1 second;* HDM - house dust mite; HS - healthy subject; NR – not reported; PC_{20} – concentration of methacholine/provocholine causing a 20% decrease in FEV_1; SCIT - subcutaneous immunotherapy; SPT - skin prick test

Table S4. The risk of bias included in a systematic review assessed using the OHAT risk of bias.

	Was administered dose or exposure level adequately randomized?	Was allocation to study groups adequately concealed?	Did selection of study participants result in appropriate comparison groups?	Were experimental conditions identical across study groups?	Were the research personnel and human subjects blinded to the study group during the study?	Were outcome data complete without attrition or exclusion from analysis?	Can we be confident in the exposure characterization?	Can we be confident in the outcome assessment?	Were all measured outcomes reported?	Were there no other potential threats to internal validity (e.g., statistical methods were appropriate and researchers adhered to the study protocol)?
Teng 2022 [17]	+	+	+	++	−	+	++	++	++	++
Peng 2022 [18]	+	+	+	++	−	++	++	+	++	+
Liu 2022 [19]	+	+	+	++	−	++	++	++	++	+
Li 2022 [20]	+	+	+	++	−	++	++	++	++	++
Jiang 2022 [21]	+	+	++	+	−	+	+	+	+	+
Chiang 2022 [22]	+	+	++	+	−	+	+	++	+	+
Zhou 2021 [23]	+	+	++	++	−	++	+	++	++	+

Wang 2021 [24]	+	+	+	+	−	++	+	+	++	+
Samra 2021 [25]	+	+	++	+	−	+	++	++	+	+
Mo 2021 [26]	+	+	++	++	−	++	++	++	++	++
Mariani 2021 [27]	+	+	+	+	−	++	++	+	++	++
Fang 2021 [28]	+	+	++	++	−	++	++	++	++	++
Zhu 2020 [29]	+	+	++	++	−	++	++	++	++	++
Fang 2020 [30]	+	+	++	+	−	++	++	++	+	++
Samra 2019 [31]	+	+	+	+	−	+	+	++	+	+
Wu 2015 [32]	+	+	+	+	−	+	+	++	++	+
Luo 2015 [33]	+	+	++	++	−	++	++	++	+	++
Qiu 2011 [34]	+	+	−	−	−	+	+	−	++	−

++ sign indicates low, + sign indicates probably low, − sign indicates probably high, −− sign indicates high risk of bias
